# Dynamic interaction between fetal adversity and a genetic score reflecting dopamine function on developmental outcomes at 36 months

**DOI:** 10.1371/journal.pone.0177344

**Published:** 2017-05-15

**Authors:** Adrianne R. Bischoff, Irina Pokhvisneva, Étienne Léger, Hélène Gaudreau, Meir Steiner, James L. Kennedy, Kieran J. O’Donnell, Josie Diorio, Michael J. Meaney, Patrícia P. Silveira, on behalf of the MAVAN research team

**Affiliations:** 1 Department of Pediatrics, Division of Neonatology, University of Toronto and the Hospital for Sick Children, Toronto, Ontario, Canada; 2 Ludmer Centre for Neuroinformatics and Mental Health, Douglas Mental Health University Institute, McGill University, Douglas Mental Health University Institute, Montreal, Quèbec, Canada; 3 Department of Psychiatry and Behavioural Neurosciences, McMaster University, Hamilton, Ontario, Canada; 4 Department of Psychiatry, University of Toronto and Centre for Addiction and Mental Health, Toronto, Ontario, Canada; 5 Child and Brain Development Program, Canadian Institute for Advanced Research (CIFAR), Toronto, Ontario, Canada; 6 Department of Psychiatry, McGill University, Montreal, Quebec, Canada; Johns Hopkins University, UNITED STATES

## Abstract

**Background:**

Fetal adversity, evidenced by poor fetal growth for instance, is associated with increased risk for several diseases later in life. Classical cut-offs to characterize small (SGA) and large for gestational age (LGA) newborns are used to define long term vulnerability. We aimed at exploring the possible dynamism of different birth weight cut-offs in defining vulnerability in developmental outcomes (through the Bayley Scales of Infant and Toddler Development), using the example of a gene vs. fetal adversity interaction considering gene choices based on functional relevance to the studied outcome.

**Methods:**

36-month-old children from an established prospective birth cohort (Maternal Adversity, Vulnerability, and Neurodevelopment) were classified according to birth weight ratio (BWR) (SGA ≤0.85, LGA >1.15, exploring a wide range of other cut-offs) and genotyped for polymorphisms associated with dopamine signaling (TaqIA-A1 allele, DRD2-141C Ins/Ins, DRD4 7-repeat, DAT1-10- repeat, Met/Met-COMT), composing a score based on the described function, in which hypofunctional variants received lower scores.

**Results:**

There were 251 children (123 girls and 128 boys). Using the classic cut-offs (0.85 and 1.15), there were no statistically significant interactions between the neonatal groups and the dopamine genetic score. However, when changing the cut-offs, it is possible to see ranges of BWR that could be associated with vulnerability to poorer development according to the variation in the dopamine function.

**Conclusion:**

The classic birth weight cut-offs to define SGA and LGA newborns should be seen with caution, as depending on the outcome in question, the protocols for long-term follow up could be either too inclusive—therefore most costly, or unable to screen true vulnerabilities—and therefore ineffective to establish early interventions and primary prevention.

## Introduction

The Developmental Origins of Health and Disease (DOHaD) concept explores the idea that variations in the quality of the early environment influence the risk for developing chronic health conditions over the life course [1, 2]. One marker of fetal adversity is poor fetal growth; being born small for gestational age (SGA) is associated with increased risk for several diseases later in life, including a wide range of metabolic [3–5] as well as mental health outcomes [6–9]. More recently, large for gestational age (LGA) newborns were also identified as being at long-term risk for developing metabolic syndrome [10] and psychopathology [11–15].

Despite the several above-described association studies, it is still challenging to clearly define which newborns are at risk for developing chronic diseases, who should be followed closely during childhood, and which are the specific risks. The classical 10^th^ percentile cut-off for birth weight at a given gestational age is somewhat arbitrary to define vulnerability in general, and may even be variable according to the evaluated outcome. Similarly, the well described “inverted U shape” association that characterizes the relationship between birth weight and later disease risk [16] suggests that the correlations between birth weight and vulnerability are not linear. It is intriguing to think that the inflection points for the inverted “U” could vary depending on the outcomes studied. In other words, the optimal birth weight for avoiding later metabolic risk may not be the same as the optimal birth weight for better developmental outcomes, for instance.

Using the classic cut-offs, SGA is associated with poorer developmental scores in children from 6 to 36 months [17–19]. A systematic review by Levine et al. identified that the most common developmental outcomes associated with poor fetal growth were motor, cognitive and language delays [17]. Reduced adaptive behavior skills have also been reported [20]. The burden becomes even more significant when acknowledging that impaired fetal growth is also associated with worse long term outcomes such as lower educational achievement and economic status in adulthood [21]. Maternal obesity, a known factor associated with both SGA and LGA births, has also been linked to behavioral difficulties and attention-deficit hyperactive disorder (ADHD) in the offspring [22]. Similarly, relationships between being born LGA and later developmental disorders have been described [23].

An interesting neurobiological target that plays a role in many of these domains is dopamine (DA). This neurotransmitter is involved in reinforcement/ reward [24], learning [25, 26] and motor development [25]. For instance, genetic variations on different components of the dopaminergic system are associated with mental health diseases such as ADHD [27], obsessive compulsive disorder [28] and schizophrenia [29]. In rodents, it was shown that a selective destruction of DA terminals or the blockade of cortical DA receptors impair motor learning, while not affecting the execution of a previously acquired skill [30]. Moreover, natural genetic variation in the number of mesocortical dopamine neurons or expression of DA-related genes in the cortex seem to explain interindividual differences in motor learning in mice [31]. There are also studies linking specific polymorphisms of the dopamine receptor type 2 (DRD2) to variation in developmental scores in children [32] as well as to verbal fluency, cognitive flexibility and creativity [33, 34], suggesting that dopamine is involved in all main domains of neurodevelopment (motor skills, language, behavioral modulation, cognition and problem solving). Interestingly, experimental studies have shown that variations in fetal growth modify DA synthesis, expression and metabolism at different structures of the mesocorticolimbic system [35–37].

Different studies have explored dopamine-related genes in a candidate-gene approach, and several examples show that genetic variation in these genes is associated with cognitive diversity. For instance, the COMT gene encodes an enzyme that regulates central dopamine catabolism; variations on this gene such as the COMT Val158Met are related to differences in working memory and high order cognitive processing [38, 39]. Genetic polymorphisms found on the dopamine type 2 receptor gene (DRD2 gene, rs1799732 [−141delC]) or its regulators (rs1800497 [Taq1A]) were linked to cognitive performance [40] and to ADHD core traits and co-morbidity [41]. Similarly, DRD4 exon III VNTR has been implicated in the development of ADHD and impulsivity [42, 43], and dopamine transporter (DAT1 gene) VNTR has been related to cognitive flexibility[44] and risk taking [45]. In this study, we aimed at using a multilocus approach, in which the genetic variation of these five important polymorphisms (rs1800497 [Taq1A], COMT Val158Met [rs4680], DRD2 rs1799732 [−141delC], DAT1 and DRD4 VNTRs) was considered at the same time. The score was calculated based on the described contribution that each gene variant has on dopamine signaling, so that hypofunctional variants received lower scores.

The objective of this study is to investigate the possible dynamism of birth weight cut-offs in defining vulnerability in different outcomes. For that, we explored an interaction between fetal adversity (SGA and LGA) and DA-relate genes (genetic score reflecting dopamine function), with gene choices based on known functional relevance to the studied outcome, as an example. Considering the important role of dopamine in multiple domains of development and the increased risk for SGA and LGA children to have abnormal developmental outcomes, we hypothesized that; a) birth weight moderates the association between the genetic score and development at 3 years of age; b) the moderating effect of birth weight on this association is specific to the evaluated domain and c) it varies with the different cut-offs defining SGA and LGA.

## Methods

Individuals were selected from a prospective birth cohort (Maternal Adversity, Vulnerability and Neurodevelopment—MAVAN) [46]. The sample included children from Montreal (Quebec) and Hamilton (Ontario), Canada. Eligibility criteria for mothers included age ≥18 years, singleton pregnancy and fluency in French or English. Mothers were excluded from the study if they had severe chronic illness, placenta previa, a history of incompetent cervix, impending delivery, or had a fetus/infant born at gestational age <36 weeks or with a major anomaly. Mother and child dyads were assessed longitudinally both at home or in a laboratory setting across the child’s development. In this study, we used data from the developmental assessment done at 36 months (see below). Approval for the MAVAN project was obtained from obstetricians performing deliveries at the study hospitals and by the institutional review boards at hospitals and university affiliates: McGill University, l’Université de Montréal, the Royal Victoria Hospital, Jewish General Hospital, Centre Hospitalier de l’Université de Montréal, Hôpital Maisonneuve- Rosemont, St Joseph’s Hospital, and McMaster University, Hamilton, Ontario, Canada. Informed consent was obtained from the parents/guardians of the participants.

### SGA and LGA definitions

Birth weight ratio (BWR) is the ratio between the observed birth weight and the sex-specific mean birth weight for each gestational age for the local population [47]. This variable was used to split the sample into three groups: SGA, adequate for gestational age (AGA) and LGA as described in the Statistical Methods. For the sake of sample description, we used the classic cut-offs of BWR 0.85 and 1.15, which defined SGA and LGA groups and represent roughly 1 SD above and below the mean population birth weight. For the main analysis, these cut-offs were changed to investigate possible group differences observable in the association between the dopamine genetic score and the outcome (see details below).

### Genetic data and multilocus score definition

Saliva samples were collected and genotyping of the DNA was performed. The ANKK1/DRD2 markers (rs1800497 [Taq1A]), COMT Val158Met (rs4680) SNP, DRD2 rs1799732 [−141delC], DAT1 and DRD4 VNTRs were amplified with polymerase chain reaction (PCR)–detailed methods are described elsewhere [48]. We followed the same approach proposed by Stice et al [49], using a multilocus genetic composite driven by the biological function. In this score, genotypes associated with putatively low DA signaling received a score of 0; those associated with high DA signaling received a score of 1; intermediate heterozygotes received a score of 0.5. Specifically, TaqIA A1/A1 [50], DRD2-141C Ins/Ins carriers [51], DRD4-7 repeat carriers [52], DAT1 10R/10R [53], and COMT Met/Met [54] genotypes were assigned a score of 0 (“low”); TaqIA A2/A2, DRD2-141C Del/Del carriers, DRD4 non 7-repeat carriers, DAT1 9/9 carriers, and COMT Val/Val genotypes were assigned a score of 1 (“high”), and DRD2-141C Ins/Del, TaqIA A1/A2, DAT 1 9/10 and COMT Met/Val genotypes received a score of 0.5. The sum of the scores resulted in a multilocus composite.

In terms of linkage disequilibrium, DAT 1 gene is located on chromosome 5, COMT gene is located on chromosome 22. The other 3 polymorphisms are from genes found on chromosome 11, but an analysis using LDlink for the two snps as well as rs762502 (a snp located at the exon 3 as a proxy for DRD4 VNTR) shows that both D prime and R squared have values close to 0, indicating independence/no linkage of alleles.

### Developmental scores

Developmental outcomes were assessed using the Bayley Scales of Infant and Toddler Development II [55]. The evaluation was performed by experienced professionals within 4 months of the time point when the child reached 36 months. Three major areas of development were used in this study: Total Behavioral Rating Scale, Motor Developmental Index (PDI) (which includes fine and gross motor subtests) and Mental Developmental Index (MDI).

### Statistical methods

To define three BWR groups, two cut-offs should be used: cut-off A defines the SGA group (subjects with BWR ≤A), and cut-off B defines the LGA group (subjects with BWR >B). Subjects with a BWR between cut-offs A and B comprise the AGA group.

Sample baseline characteristics of the three groups (SGA, AGA, LGA) were compared using ANOVA test for continuous data and chi-square test for categorical variables. All subjects with BWR ≤0.85 were considered as SGA group, subjects with BWR > 1.15 were considered as LGA, and subjects with BWR in between 0.85 and 1.15 were referred to as AGA.

**Fig 1:**
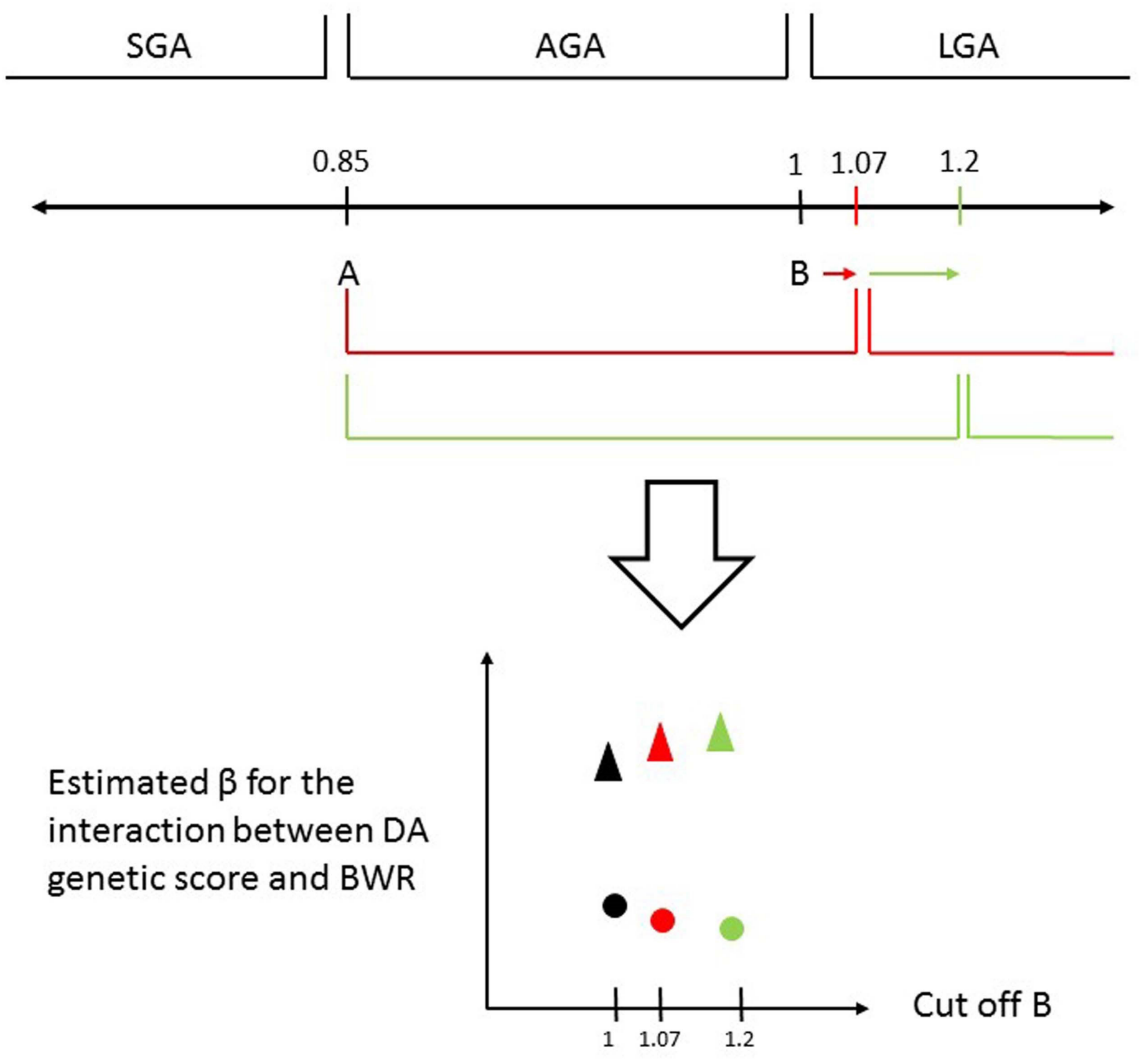
Categorization of the BWR into three groups and levels of significance for the interaction between BWR and the dopamine genetic score. Groups (SGA, AGA and LGA) are categorized according to different cut-offs (A and B). Group II was used as the reference group (AGA) for all comparisons. The graph depicts the result for a fixed cut-off A and changing cut-off B. Each dot in the plot corresponds to the difference in the estimated β for DA multilocus between SGA and AGA (circles) or LGA and AGA (triangles).

Fig 1 is a graphical representation of the scheme used to categorize BWR into three groups. For the main analysis, fixing a low cut off A at a certain value (varying from 0.7 to 1.0), we iterated the high cut-off B (from 1.01 to 1.2) each time performing a linear regression analysis to investigate if the categorized BWR moderates the association between the dopamine genetic score and each one of the three Bayley domains. Co-variates in every linear regression are categorized BWR, dopamine genetic score, their interaction and gender.

Data were analyzed using the Statistical Package for the Social Sciences (SPSS) 22.0 software (SPSS Inc., Chicago, IL, USA) and R[56]. Significance levels for all measures were set at p<0.05.

## Results

There were 251 individuals in the sample. Table 1 depicts the baseline characteristics, and no significant differences were found between the BWR groups.

**Table 1:** Baseline characteristics.

Sample characteristics	SGA (n = 52)	AGA (n = 178)	LGA (n = 21)	*P* value
Females (%)^a^	27 (51.9%)	85 (47.8%)	11 (52.4%)	0.83
Maternal age at birth (%)^b^	29.49(5.02)	30.67(4.64)	30.20(4.00)	0.28
Exclusive breastfeeding (weeks)^b^	25.85(18.81)	27.68(19.35)	24.89(17.63)	0.73
Maternal smoking during gestation (%)^a^	9 (20.5%)	17 (10.7%)	0 (0%)	0.07
Family income below LICO (%)^a^	9 (17.3%)	27 (16.4%)	2 (10%)	0.74
Diabetes since the beginning of the pregnancy (%)	1 (1.92%)	5 (2.81%)	1 (4.76%)	0.63
Number of full weeks of gestation	39.27 (1.21)	39.21 (1.17)	38.67 (1.32)	0.12
C-section (%)	9 (17.3%)	33 (18.5%)	8 (38.1%)	0.09

^a^Chi-square test and ^b^ANOVA test.

Data are expressed as Mean(SD) or number of participants (percentages). SGA = small for gestational age, AGA = adequate for gestational age, LGA = large for gestational age, LICO = Low Income Cut Off [57]. Small differences in totals are due to missing data.

Table 2 shows the genotype distribution for each gene, with the Hardy-Weinberg criteria met in all cases.

**Table 2:** Genotype distribution in the study sample.

Gene	Distribution	H-W equilibrium (*P* value)
DAT1 VNTR	10/10 (133, 53%); 9/10 (96, 38%); 9/9 (22, 9%)	0.44
DRD2 141C (rs1799732) BstNl	Ins/Ins (189, 75%); Ins/Del (57, 23%); Del/Del (5, 2%)	0.77
DRD4 VNTR	7R homozygous (8, 3%); 7R heterozygous (82, 33%); non-7R/non-7R (161, 64%)	0.53
Taq IA (rs1800497)	A1/A1 (12, 5%); A1/A2 (74, 29%); A2/A2 (165, 66%)	0.33
COMT (rs4680)	A/A (58, 23%); A/G (131, 52%); G/G (62, 25%)	0.48

Criteria for Hardy Weinberg Equilibrium were met for the five genes.

Table 3 describes the mean scores for the different Bayley domains in the three BWR groups. We show in Table 3 that, when using the classic cut-offs (0.85 and 1.15), there were no statistically significant differences observed between the groups in the three domains of Bayley; moreover, there were no significant interactions between the neonatal groups and the dopamine genetic score [Total Behavioral scale: estimated **β** = − 0.47, p = 0.21 for SGA vs AGA; estimated **β** = 0.22, p = 0.89 for LGA vs. AGA; PDI: estimated **β** = 2.3, p = 0.36 for SGA vs AGA; estimated **β** = − 6.29, p = 0.08 for LGA vs. AGA; MDI: estimated **β** = 1.16, p = 0.6 for the comparison between SGA vs AGA and estimated **β** = -3.62, p = 0.23 for LGA vs. AGA].

**Table 3:** Mean outcome measures of the Bayley tests at 36 months.

Outcome	SGA	AGA	LGA	*P* value
Total Behavioral Scale	122.44(6.25)	123.22(6.15)	125.19(4.39)	0.22
Motor Developmental Index (PDI)	99.04(11.50)	100.99(12.77)	103.67(14.16)	0.35
Mental Developmental Index (MDI)	96.96(11.50)	98.47(10.43)	99.10(10.41)	0.63

Data are expressed as Mean (SD). There were no statistically significant differences observed between the groups.

**Fig 2:**
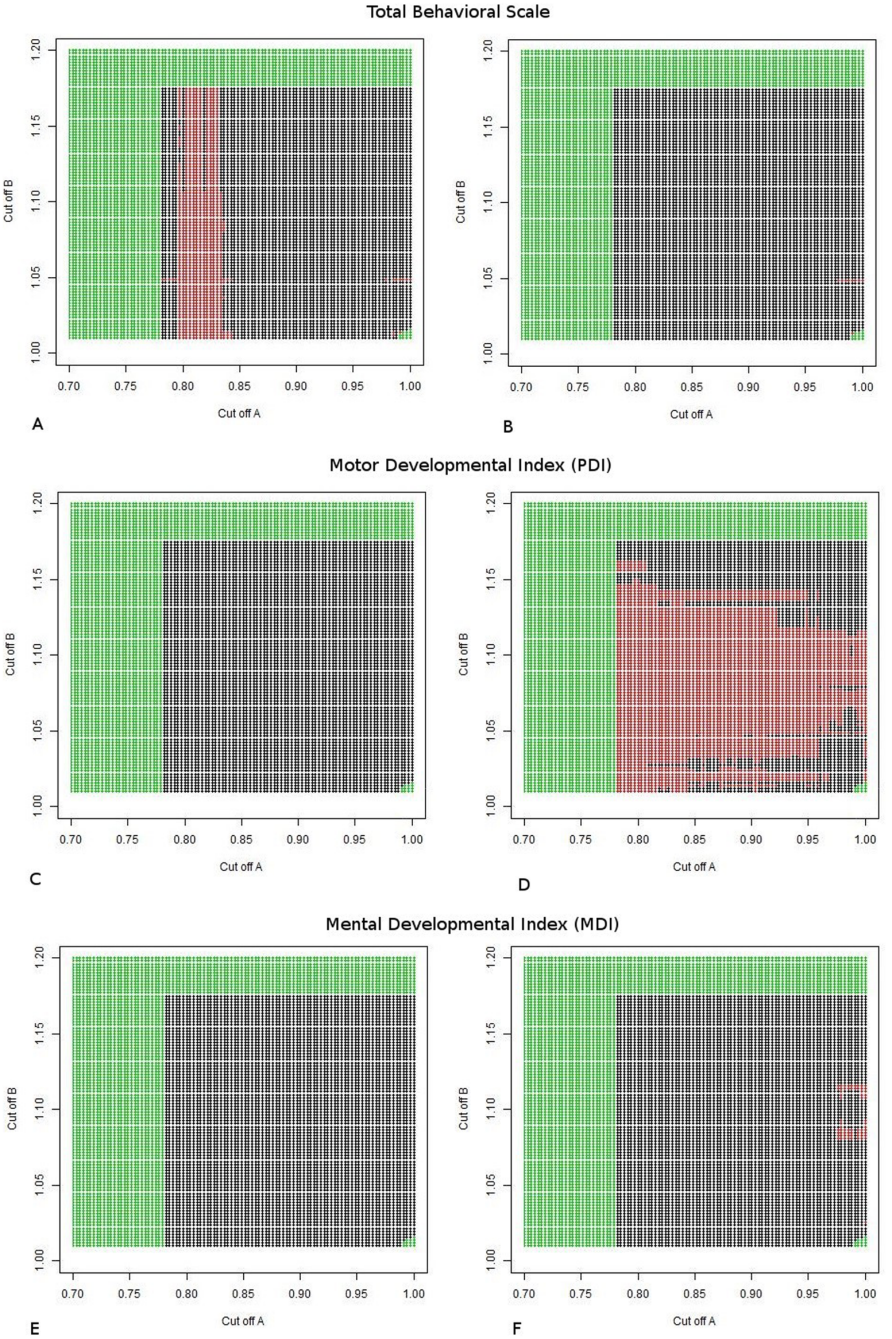
Estimated β for dopamine multilocus score. A range of SGA cut-offs is shown on the x axis, and LGA cut-offs are depicted on the y axis. Black signs show interactions that are not significant (p> = 0.05), red signs represent interactions that are statistically significant (p<0.05) and green display the regions where the comparison included a group with less than 15 participants. A and B: Total Behavioral Score; C and D: Motor Developmental Index (PDI); E and F: Mental Developmental Index (MDI) from Bayley. A, C and E are comparisons between SGA and AGA, while B, D and F are comparison between LGA and AGA.

However, when taking different BWR cut-offs, varying from 0.7 to 1.0 to define the SGA group or from 1.01 to 1.2 to define the LGA group, we observe certain significant interactions for specific outcomes (see Fig 2, **S# Videos and S# Figs**).

The different graphs on Fig 2 plot a range of SGA cut-offs on the x axis, and LGA cut-offs on the y axis, depicting the level of significance for the interaction between BWR and the dopamine genetic score if those cut offs were chosen. Black signs show interactions that are not significant (p> = 0.05), red signs represent interactions that are statistically significant (p<0.05) and green display the regions where the comparison included a group with less than 15 participants.

Fig 2A/2B, S1 Video and S1 Fig, show the results for the Total Behavioral Score from Bayley. We can see that while there were no significant interactions between BWR and the dopamine genetic score when comparing LGA to AGA (2B), there was a diverse range of cut-offs defining the SGA group that represent significant differences between SGA and AGA for the effect of dopamine genetic score on the outcome (2A). In other words, if any cut-off within the “red” range is taken to define the SGA group, there will be a significant interaction between BWR and the dopamine genetic score on the Total Behavioral score. For example, if taking 0.8 as the cut-off “A” (children with BWR ≤0.8 are considered SGA) and 1.1 as the cut-off “B” (children with BWR between 0.8 and 1.1 are AGA and higher than 1.1 are LGA), as the dopamine genetic score increases (representing higher dopaminergic function), the Total Behavioral Score decreases in the SGA group [estimated **β** = − 4.21, p = 0.008], but the slopes for both AGA [estimated **β** = − 0.79, p = 0.16] and LGA groups [estimated **β** = -1.57, p = 0.18] are not statistically significant.

Fig 2C/2D, S2 Video and S2 Fig show the results for the Motor Developmental Index (PDI) from Bayley. We see that while there were no significant interactions between BWR and the dopamine genetic score when comparing SGA to AGA (2C), there was a range of cut-offs defining the LGA group representing significant differences between LGA and AGA for the effect of dopamine genetic score on the outcome (2D). Specifically, if any cut-off within the red range is taken to define the LGA group, there will be a significant interaction between BWR and the dopamine genetic score on PDI. Using the same example, when taking 0.8 as the cut-off “A” and 1.1 as the cut-off “B”, as the dopamine genetic score increases, PDI decreases in the LGA group [estimated **β** = -6.51, p = 0.009], but the slopes for both AGA [estimated **β** = 1.25, p = 0.28] and SGA groups [estimated **β** = -1.93, p = 0.55] are not statistically significant.

Fig 2E/2F, S3 Video and S3 Fig show the results for the Mental Developmental Index (MDI) from Bayley. For any cut-offs chosen as “A” and “B”, there were no statistically significant interactions found between BWR and the dopamine genetic score. Although a few statistically significant points are seen in the figures, these are most likely due to artifact than to true results.

The videos show the different “B” cut-offs in the x axis for a fixed cut-off “A” in each frame. In the y axis, the estimated beta coefficients for the interaction between BWR and the dopamine genetic score are displayed. AGA group was considered the reference group in all the analysis. Circles represent the difference in the dopamine genetic score slopes between SGA and AGA groups, and the triangles display the difference between LGA and AGA groups. Video 1 shows data for Total Behavioral Score, video 2 for PDI and video 3 for MDI scores. It is possible to observe that, according to our hypothesis, birth weight moderates the association between the multilocus genetic score reflecting dopaminergic function and development at 3 years of age, but this is limited to and variable across the specific Bayley domains, as well as with the different cut-offs defining SGA and LGA.

## Discussion

This study exemplifies how limited is the concept of long-term vulnerability defined by standard birth weight cut offs currently used in Pediatrics. More specifically, we described here that birth weight moderates the association between a genetic score reflecting dopaminergic function and development at 3 years of age, but this is specific to certain developmental domains, and varies according to the different cut-offs defining SGA and LGA.

For a certain range of cut-offs on BWR, the results showed significant differences comparing SGA and AGA on the Total Behavioral scale. In addition, LGA and AGA groups significantly differed in the Motor Developmental Index using a diverse range of “B” cut offs. These findings agree with the literature showing that both low and high birth weight children have increased risk for several conditions during the life course [3–14], but not necessarily the same poor outcomes are expected for both groups.

The novelty aspect of the current study lies in the fact that, using the classic cut-offs to define SGA and LGA, these interactions would not be perceived. This highlights the discrete line that separates those at risk versus those that are not, as well as the specificity of this line according to the studied outcome. From a clinical standpoint, this means that there is a fine balance between a) being too inclusive to select a wide variety of children for close follow up during childhood, increasing the health care costs, the burden of repeated assessments as well as the stigma of being “fragile” or b) being too strict in the definition of the group to be followed, and therefore missing the opportunity for screening those at risk and for establishing preventive or intervention measures. Although we do not provide a final definition, we highlight the importance of research in this field and the urge for the development of algorithms to be applied very early in life—potentially at birth—that could predict risk for disease based on prenatal history.

It is important to mention that studies involving gene versus environment interactions can be very challenging. It is hard to deal with the multitude of variation in the environment (e.g. home, school, society) and how these several “layers” influence the risk for disease [58]. In addition, single candidate gene studies are obviously limited considering the whole genome richness, therefore methods involving a pathway-based strategy, like the one used in this study, may be physiologically more relevant.

The significant interactions between fetal growth and the DA multilocus on the Motor domain of the Bayley II scores are not surprising considering that DA is in close relationship to movement disorders such as Parkinson and Huntington’s diseases [59]. Similarly to what occurs in these diseases, unbalances in basal ganglia pathways, which are largely regulated by DA, are likely implicated in the more subtle changes seen in gross and fine motor skills evaluated through the Bayley-II.

The association between DA and behavioral aspects is also clear in the literature. There is consistent evidence that some dopamine gene polymorphisms are involved in the etiology of ADHD, one of the most prevalent childhood psychiatric disorder [60, 61]. The DA hypothesis is supported by animal, pharmacological, brain imaging and genetic studies [62]. Alterations in the mesocorticolimbic pathway correlate with impulsivity [63] and variations in selective attention [64], while the nigrostriatal pathway is associated with hyperactivity [65].

The DA system seems to be particularly vulnerable to variations in the environment [58, 66]. Research suggests that genes may have been naturally selected as a form to bet-hedge against an uncertain future [67], both for conditional and fixed health strategies. In the case of DA-related genes and specifically in the example illustrated in our study, fetal growth could signal the quality of the uterine environment, with both aberrant extremes (poor or excessive growth) seen as unfavorable conditions and therefore leading to worse outcomes [68].

One of the limitations of this study lies on the fact that we were not able to precise which cut-offs would be optimal to define developmental vulnerability according to the dopamine genetic score. However, this would be a rather specific information, pending external validity. In addition, our multilocus score considers that “risk” has the same weight across the different polymorphisms, as opposed to characterizing them in terms of a particular odds ratio for a certain outcome. One of the reasons for that is to consider that 2 out of the 5 genetic polymorphisms from our score are VNTRs, and these are not included in Genome Wide Association Studies (GWAS). As a long list of scientific evidence has shown that the genetic variation involving these polymorphisms plays an important role in modifying neurocognitive outcomes, we assume that their relevance cannot be denied [69]. In addition, as highlighted above, dopamine genes could function as “plasticity genes”, having their association with the outcome changing directionality in terms of risk/protection according to variations in the environment (in our case, the fetal environment)[58, 66]. We believe that message of awareness regarding the dynamic relationship between different birth weight cut-offs and long-term risk, as well as about the specificity of this relationship to certain outcomes is relevant and could be applicable in other contexts.

In summary, the classic birth weight cut-offs to define SGA and LGA newborns should be seen with caution, as depending on the outcome in question, the protocols for long-term follow up could be either too inclusive—therefore most costly, or unable to screen true vulnerabilities—and therefore ineffective to establish early interventions and primary prevention. Our study suggests that the established cut-offs should not be used blindly; we favor a personalized approach to pediatric follow up, considering the different aspects of the child’s history (ex.: fetal growth, birth and neonatal trajectory, family history, current development, etc.) as well as highlight the importance of close and repeated developmental assessments during childhood.

## Supporting information

S1 Fig Still image of the video showing the results for the Total Behavioral Score. (TIF)

S2 Fig Still image of the video showing the results for the Motor Developmental Index. (TIF)

S3 Fig Still image of the video showing the results for the Mental Developmental Index. (TIF)

S1 Video Video showing the results for the Total Behavioral Score. (MP4)

S2 Video Video showing the results for the Motor Developmental Index. (MP4)

S3 Video Video showing the results for the Mental Developmental Index. (MP4)
