# Supplementary figures and images for: Dynamic interaction between fetal adversity and a genetic score reflecting dopamine function on developmental outcomes at 36 months
Source: PLoS One. 2017 May 15;12(5):e0177344. doi: 10.1371/journal.pone.0177344 (PMC5432105; doi:10.1371/journal.pone.0177344)

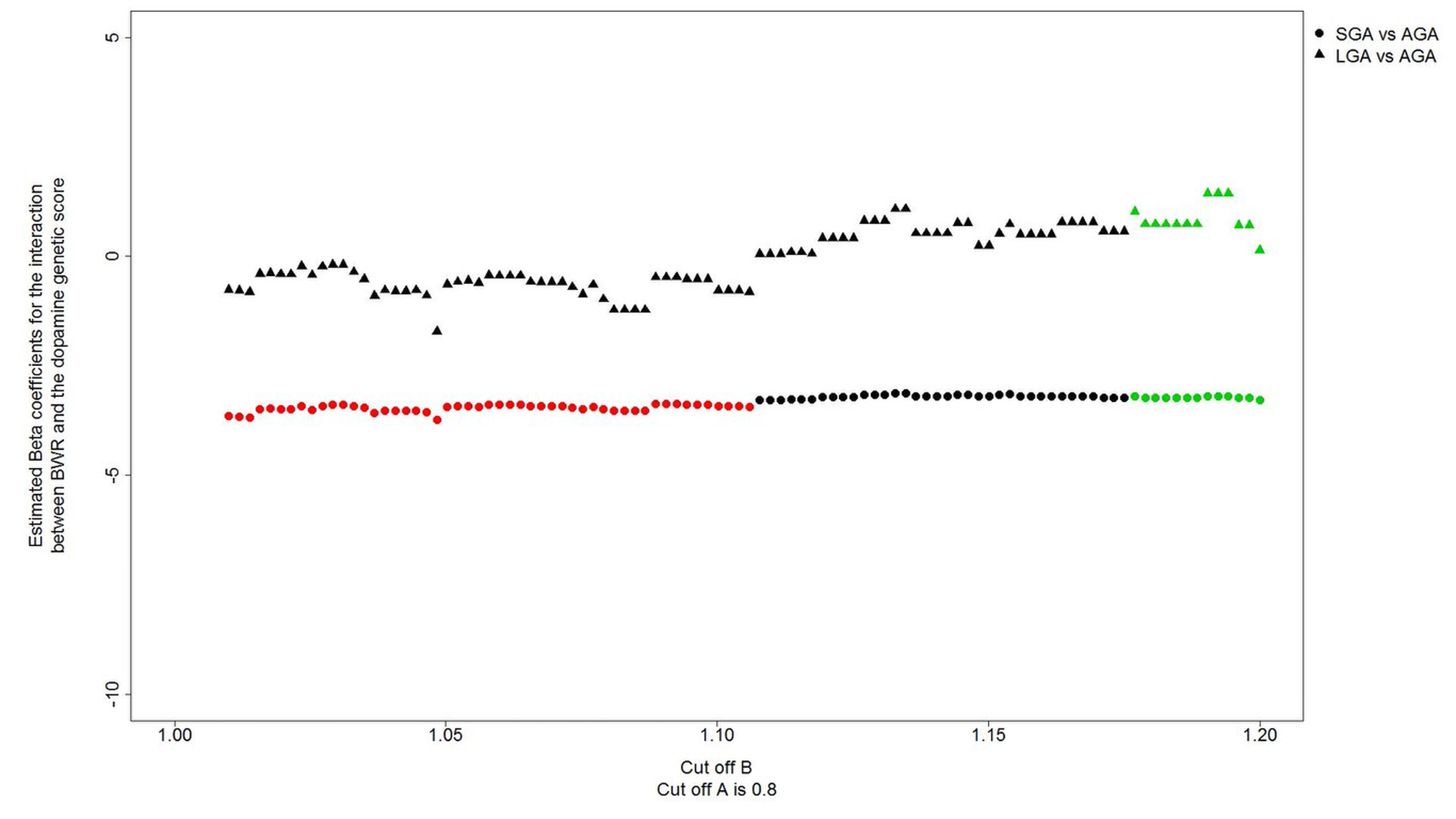

Supplement: S1 Fig — (TIF) [file pone.0177344.s001.tif]

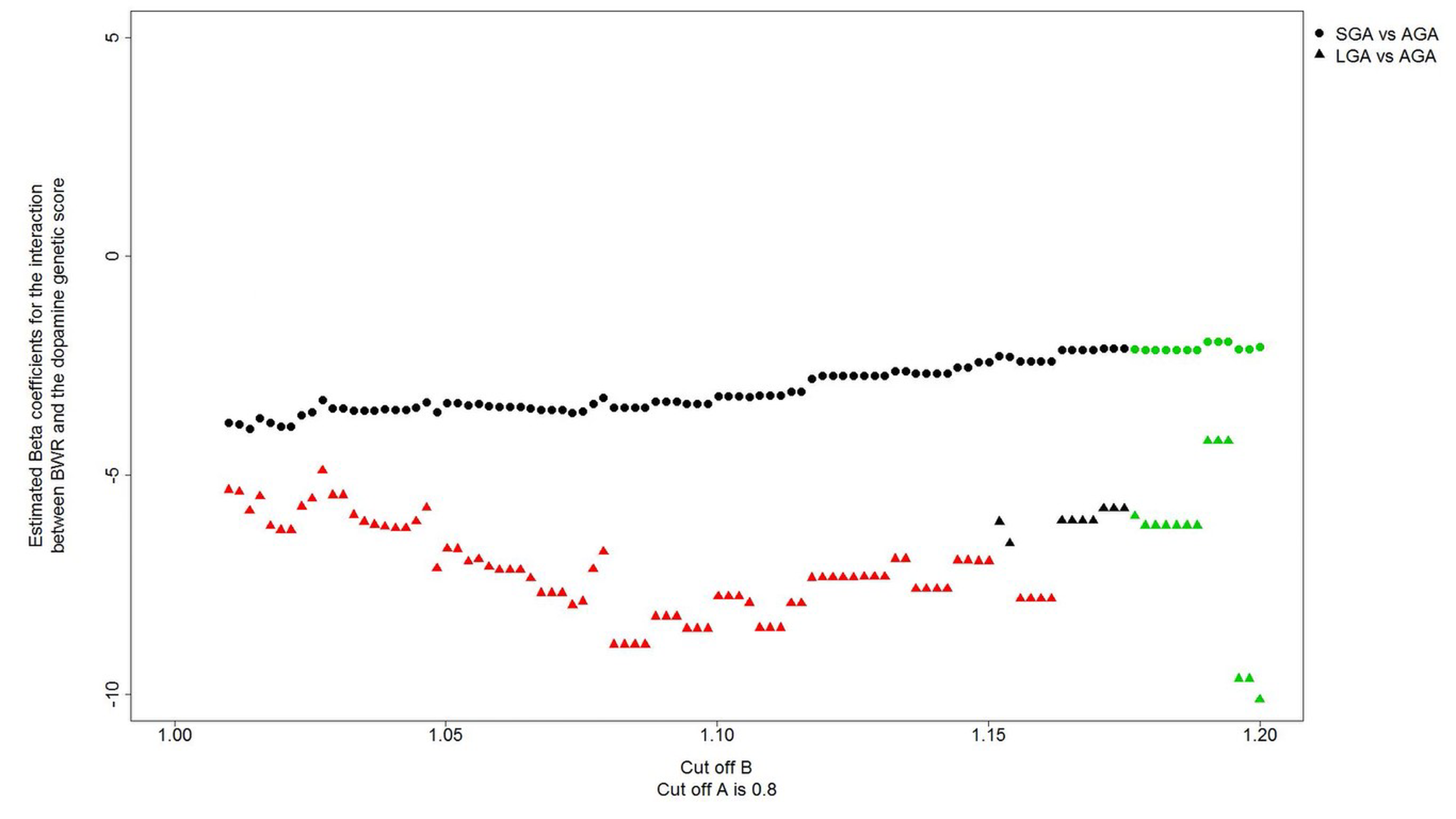

Supplement: S2 Fig — (TIF) [file pone.0177344.s002.tif]

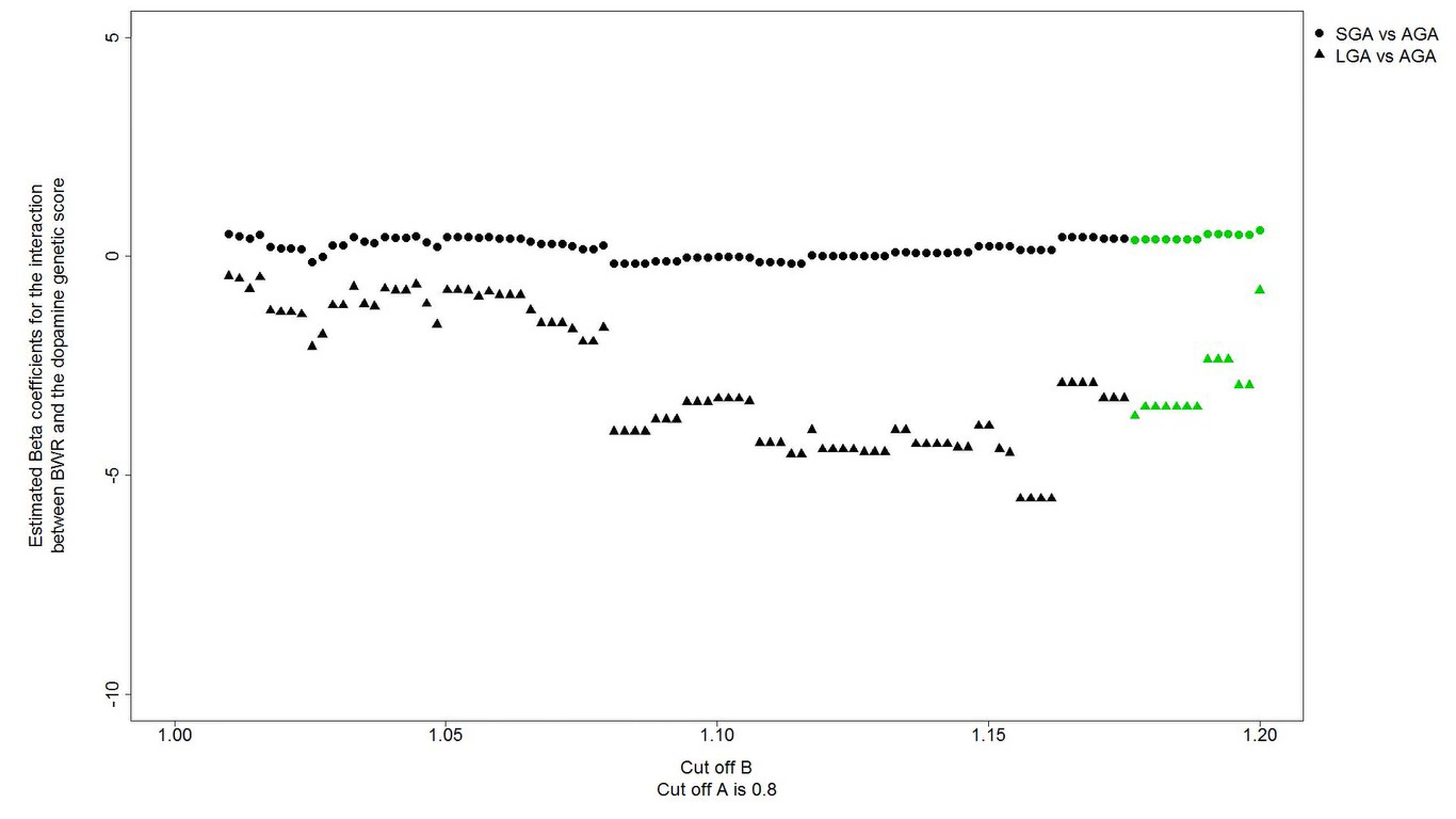

Supplement: S3 Fig — (TIF) [file pone.0177344.s003.tif]
